# Serum IgA Antibodies Specific to *M. leprae* Antigens as Biomarkers for Leprosy Detection and Household Contact Tracking

**DOI:** 10.3389/fmed.2021.698495

**Published:** 2021-08-10

**Authors:** Kyssia Karen de Paiva e Silva, Erick Esteves de Oliveira, Carolina Martins Moreira Elias, Ingrid Estevam Pereira, Roberta Olmo Pinheiro, Euzenir Nunes Sarno, Malcolm Scott Duthie, Henrique Couto Teixeira

**Affiliations:** ^1^Department of Parasitology, Microbiology and Immunology, Institute of Biological Sciences, Federal University of Juiz de Fora, Juiz de Fora, Brazil; ^2^Leprosy Laboratory, Oswaldo Cruz Institute, Oswaldo Cruz Foundation, Rio de Janeiro, Brazil; ^3^HDT Biocorp, Seattle, WA, United States

**Keywords:** leprosy, diagnosis, IgA, biomarker, NDO-HSA, LID-1, ROC analysis, household contact

## Abstract

Leprosy remains endemic in several developing countries, such as India and Brazil, in part due to delayed diagnosis that facilitates ongoing transmission. Although immunoglobulins against several *Mycobacterium leprae* antigens have been indicated for the early diagnosis, and IgA participates in the early stages of leprosy and in subclinical infection, relatively little research has examined anti-M. leprae IgA responses. Here, we investigated serum IgA reactivity against NDO-HSA, LID-1 and NDO-LID, in paucibacillary (PB) and multibacillary (MB) patients and their household contacts, using enzyme-linked immunosorbent assay (ELISA). Diagnostic accuracy of each ELISA was evaluated by receiver operating characteristic (ROC) curve analysis. Our data reveal elevated IgA serum levels against the three *M. leprae* specific antigens in MB patients, whereas IgA reactivity in PB patients was increased only to NDO-HSA. Further, MB and PB household contacts displayed higher IgA reactivity to NDO-HSA than non-endemic controls. Our data suggest measurement of serum IgA against NDO-HSA as an additional tool in the diagnosis and classification of the disease, with potential utility for household contact follow-up.

## Introduction

Leprosy, also known as Hansen's disease, is a chronic granulomatous disease that mainly affects the skin and peripheral nerves and, among infectious diseases, is the leading cause of physical disabilities and stigma (1, 2). *Mycobacterium leprae*, the etiological agent of leprosy, is an intracellular bacterium with tropism for macrophages and Schwann cells (1). The disease is likely transmitted via droplets, from the nose and mouth, during close and frequent contact with untreated cases (3). Most of the infected population remains free of the disease, while a subset of infected individuals develops clinical symptoms that are associated with the immunity of the host (2, 4). Therefore, early detection of *M. leprae* infection, before the clinical manifestations, is paramount to reduce the transmission (5).

For treatment purposes and according to clinical and microbiological findings, leprosy patients are classified into two major groups: paucibacillary (PB), those with up to five skin lesions and/or an affected nerve trunk, and multibacillary (MB), those with more than five skin lesions and/or more than one affected nerve trunk. In addition, patients whose skin-smear exam tests positive are classified as MB regardless of the number of lesions (3).

The diagnosis of leprosy is hampered by the broad spectrum of clinical forms dictated by the host's immune response to *M. leprae*, ranging from disseminated infection to a self-limited form of the disease, with lack of effective testing available to detect asymptomatic infection or predict disease progression (6). In this context, the search for immune biomarkers of infection has been focused upon specific antibodies. Although serology is not used routinely, it has been widely explored in research studies since the discovery of the phenolic glycolipid antigen I (PGL-I), a cell wall antigen of *M. leprae* which induces the production of specific IgM response detected in patient serum (6). Despite nearly all MB leprosy patients being positive for anti-PGL-I IgM responses, most PB leprosy patients do not develop detectable antibody levels against PGL-I (5). The increased humoral response in MB patients, however, fails to eliminate *M. leprae*, and thus favor disease progression and bacillary spread (7, 8).

Besides serology for PGL-I, other *M. leprae* antigens have shown immunodiagnostic potentials, such as native lipoarabinomannan (LAM) antigen and the secreted proteins Ag85 (ML2028) and CFP-10 (ML0050) (4, 5). In addition, IgM and IgG antibody responses directed against *M. leprae*-specific recombinant proteins have also been tested in serologic assays. A previous study from our group identified marked increases in serum IgM antibodies against NDO-HSA (a conjugate formed by natural octyl disaccharide bound to human serum albumin) and IgG antibodies against LID-1 (the fusion protein product of the ml0405 and ml2331 genes), as well as elevated IgM/G antibodies against NDO-LID (a combination of LID-1 and NDO) in MB patients, but not in PB patients (9). In addition, a selective increase in IgG1 and IgG3 antibodies against LID-1 and NDO-LID was detected only in MB patients, indicating potential of improvements in serodiagnosing MB leprosy patients (9).

In order to reduce transmission, efforts have focused on detecting *M. leprae* infection before the onset of clinical manifestations. Duthie et al. suggest that anti-NDO-LID responses can diagnose and monitor leprosy patients, detecting a significant number of patients in the earlier stages of disease development (10). Quiong-Hua et al. demonstrate that anti-LID-1 responses may be a tool for early diagnosis in household contacts of MB leprosy patients (11). In addition, anti-LID-1 and anti-NDO-LID responses are more effective than anti-NDO-HSA for the detection of MB leprosy and for the identification of individuals with subclinical infection (12).

It has been suggested that IgA participates in early stages of leprosy disease and in subclinical infection (13, 14), however, few reports have addressed anti-*M.leprae* IgA responses. IgA may protect against mycobacterial infections of the respiratory tract through the blockage of pathogen entrance and/or modulating the pro-inflammatory responses (15). Moreover, IgA is being considered as an alternative or complementary biomarker in the diagnosis of pathologies such as toxoplasmosis and acute dengue (16, 17). Demonstrating a good correlation between salivary anti-PGL-I IgA and IgM levels in MB patients, Nagao-Dias et al. (2007) showed that anti-PGL-I IgA and IgM salivary antibodies are significantly higher in MB patients compared to normal controls, but not when compared to PB patients (18).

The importance of IgA for mucosal host immunity, especially in the respiratory and digestive tracts, is well established, although its role in systemic circulation is still unclear (19). In the present work, we assessed serum IgA reactivity to NDO-HSA, LID-1 and NDO-LID in patients with paucibacillary (PB) and multibacillary (MB) leprosy and their household contacts, using enzyme-linked immunosorbent assay (ELISA). Diagnostic accuracy of each ELISA was evaluated by receiver operating characteristic (ROC) curve analysis.

## Materials and Methods

### Study Population

Leprosy patients (*n* = 37) and household contacts (*n* = 40) were recruited at the Souza Araújo ambulatory in Oswaldo Cruz Foundation, Rio de Janeiro (FIOCRUZ-RJ, Brazil). Patients were characterized as paucibacillary (PB/*n* = 19), when presenting five or less skin lesions and negative bacilloscopy, or multibacillary (MB/*n* = 18) when presenting with more than five lesions and/or positive bacilloscopy, as described by the operational classification adopted by the World Health Organization (3). Patients were further characterized according to the Ridley- Jopling classification system of clinical manifestations (Table 1) (20). The household contacts, defined as people who lived for at least five years with leprosy patients before the diagnosis, were divided into the paucibacillary household contacts (PB-C/*n* = 20) and multibacillary household contacts (MB-C/*n* = 20). Two control groups, without prior history of mycobacterial disease, were also studied: the non-endemic controls (NEC/*n* = 20) consisted of individuals from Juiz de Fora – MG – Brazil, a non-endemic region; and the endemic controls (EC/*n* = 18) recruited in Rio de Janeiro, after undergoing dermatoneurological examinations. Patients with comorbidities such diabetes, hepatitis, syphilis, diseases caused by other mycobacteria, patients co-infected with the human immunodeficiency virus, treated patients and relapse cases were excluded. All patients and controls gave informed consent for blood sampling after written information was provided. This study was approved by the Ethical Committee of the Oswaldo Cruz Institute (protocol: 1.896.348).

**Table 1:** Characteristics of the study participants.

**Groups**	***n***	**Sex (M/F)**	**Age (mean)**	**R-J Classification**
Paucibacillary patients (PB)	19	7/12	15–72 (50.2)	19 BT
Multibacillary patients (MB)	18	14/4	11–73 (43.0)	6 BL/12 LL
Paucibacillary contacts (PB-C)	20	9/11	15–67 (37.4)	–
Multibacillary contacts (MB-C)	20	9/11	13–60 (37.6)	–
Endemic controls (EC)	18	3/15	20–49 (26.4)	–
Non-endemic controls (NEC)	20	5/15	20–56 (29.6)	–
Total	115	47/68	11–73 (37.3)	–

*R-J, Ridley-Jopling classification; BT, Borderline tuberculoid; BL, Borderline lepromatous; LL, Lepromatous form*.

### Detection of Antigen-Specific IgA by Enzyme-Linked Immunosorbent Assay (ELISA)

Polystyrene 96-well microplates were coated overnight with NDO-HSA, LID-1 and NDO-LID antigens (2 μg/mL) diluted in 0.06 M carbonate buffer (pH 9.6) solution (50 μL per well). Wells were then washed with phosphate-buffered saline (PBS) containing 0.05% Tween 20 (PBS-T) and blocked with 1% bovine serum albumin (BSA) in PBS-T for 1 h at 37°C. Serum samples, previously collected and stored at −20°C, were thawed, diluted 1:20 in PBS-T containing 0.1% BSA and added in duplicates (50 μL per well). After incubation at 37°C for 1h, plates were washed in PBS-T before adding aliquots of 50 μL per well of rabbit anti-human IgA α-chain specific Peroxidase antibody (1:2,000) (Sigma Aldrich SAB3701236, St. Louis, Missouri, EUA), conjugated with horseradish peroxidase (HRP). After 1h of incubation at 37°C, wells were washed with PBS-T and a substrate solution containing 0.5 mg/mL ortho-phenylenediamine in sodium citrate buffer, pH 5.0, and 0.03% H2O2 was used. The reaction was terminated with 2N H2SO4 and the optical density measured at 492 nm (Spectramax-190, Molecular Devices, Sunnyvale, CA, USA). The results were expressed as the average of the optical density (OD) of the replicates.

### Statistical Analysis

To obtain the accuracy values (sensitivity and specificity), the MedCalc Statistical (Version 5.00.020, Brussels, Belgium) was used to set a Receiver Operating Characteristic (ROC) Curve. In addition, the area under the ROC curve was used to compare the performance of the tests. In the comparisons, a *p* < 0.05 was considered statistically significant. The presented data did not follow a normal distribution, as determined by the Shapiro-Wilk test. Therefore, means were compared using Kruskal-Wallis test followed by Dunn's test, *p* < 0.05. The statistical analyses were performed using the software GraphPad Prism 5.0 (GraphPad Software, San Diego, CA, USA).

## Results

### Multibacillary Patients Present With High IgA Reactivity to Different *M. leprae* Antigens

Despite of its important role in mucosal responses, it is often overlooked that IgA is the second most prevalent antibody class in the blood (21, 22). However, the use of serological tests based on IgA to provide leprosy diagnosis is still incipient.

**Figure 1:**
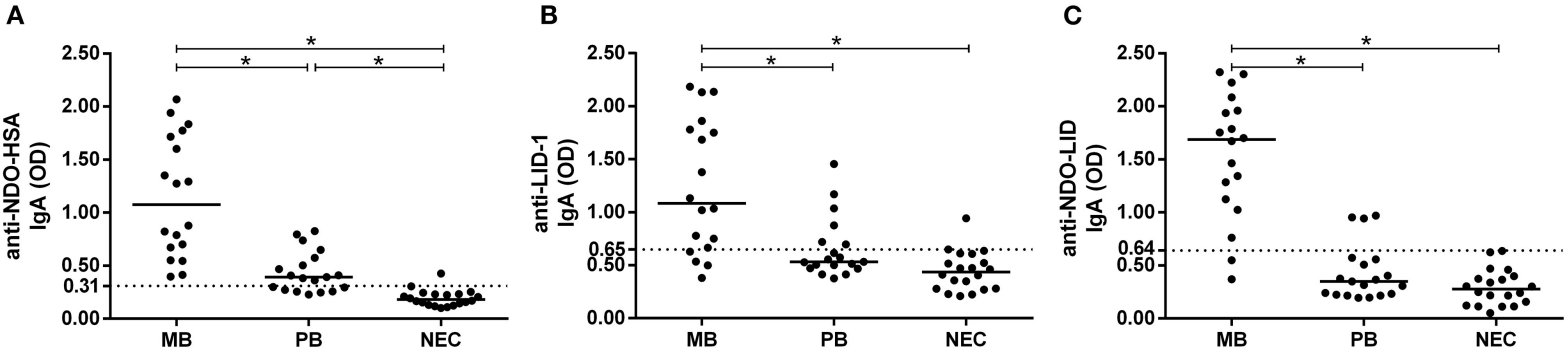
Antigen-specific IgA responses in multibacillary (MB) and paucibacillary (PB) leprosy. Levels of IgA antibodies against **(A)** NDO-HSA, **(B)** LID-1, and **(C)** NDO-LID in serum of MB (*n* = 18) and PB (*n* = 19) leprosy patients and in non-endemic controls (NEC; *n* = 20) were measured by ELISA. The cut-off point (dashed line) was established by the ROC curve while the horizontal bars represent median. * = *P* < 0.05.

In the present study, the multibacillary group (MB) presented high IgA reactivity to NDO-HSA, LID-1 and NDO-LID when compared to both the non-endemic control group (NEC) and to the paucibacillary group (PB) (Figure 1). Although lower, the IgA response to NDO-HSA in the PB group was also greater than that observed in NEC, reaching 63% of seropositivity, whereas IgA reactivity to LID-1 and NDO-LID groups was not significantly increased in the PB group vs. NEC.

### NDO-HSA Performance Overcome the Other Antigens

To further assess the performance of NDO-HSA, LID-1 and NDO-LID antigens in MB and PB patients, the IgA responses were plotted in ROC curves to provide sensitivity and specificity values, as well as the area under the curve (AUC) (Table 2). For the MB groups, the AUC for both NDO-HSA [AUC = 0.994] and NDO-LID [AUC = 0.978] were slightly higher than those observed for LID-1 [AUC = 0.917]. For the PB groups, the AUC was significantly higher only for NDO-HSA [AUC = 0.937].

**Table 2:** Sensitivity and specificity of NDO-HSA, LID-1 and NDO-LID antigens in IgA-based serodiagnosis of MB and PB leprosy.

**Antigens**	**Clinical form**	**Cutoff^a^**	**Sensitivity^b^ (%)**	**Specificity^b^(%)**	**Area under the curve**
NDO-HSA	MB	0.31	100	95	0.994
	PB	0.244	95	85	0.937
LID-1	MB	0.65	78	95	0.917
	PB	0.412	95	50	0.746
NDO-LID	MB	0.64	89	100	0.978
	PB	0.158	99	30	0.642

a *, cutoff, sensitivity and specificity data were determined based on the analysis of receiver operating characteristic (ROC) curves*;

b *, the values of sensitivity and specificity were determined according to the point of the ROC curve nearest to the point of sensitivity and specificity equal to 100%*.

The ROC curve analysis showed that at the optimal cut off (i.e., the point located nearest to the left upper corner of the ROC curve Cartesian space) NDO-HSA-specific IgA levels provided a sensitivity of 100% with a specificity of 95% for the MB group and a sensitivity of 95% with a specificity of 85% for PB. LID-1 and NDO-LID showed higher sensitivity for the PB group (95 and 99%, respectively) but with lower specificity (50 and 30%, respectively) (Table 2). Altogether, the data shows that detecting IgA against NDO-HSA provides high serodiagnostic performance regardless of the clinical form of leprosy.

### Endemic Controls and Household Contacts Display Increased Reactivity to NDO-HSA

Household contacts, due to their high exposure, represent a risk group for leprosy development. IgA reactivity against the NDO-HSA, LID-1 and NDO-LID antigens in household contacts of either MB or PB were evaluated and compared to the endemic (EC) and non-endemic (NEC) controls. The contacts of the different leprosy forms were separated to reveal any distinct profiles between these groups.

**Figure 2:**
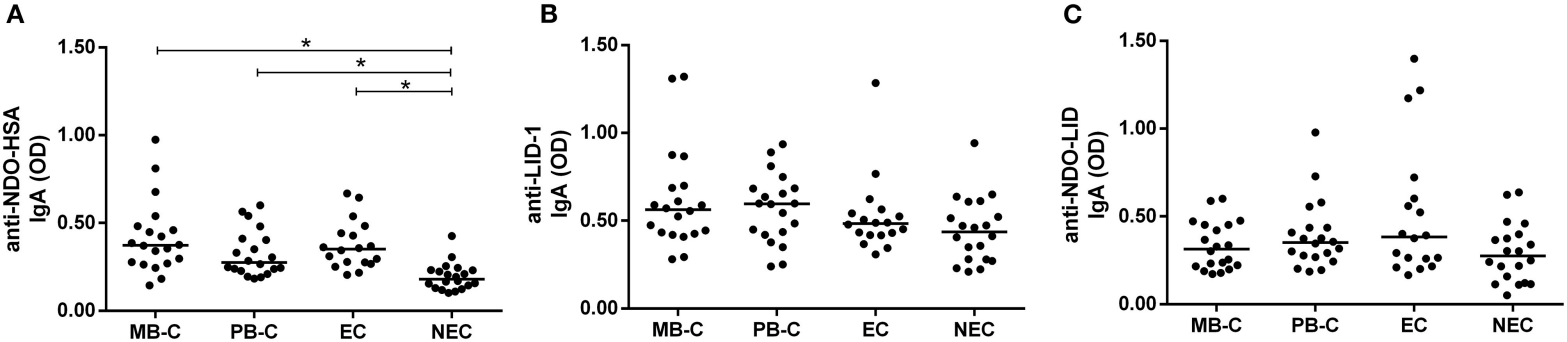
Serum IgA responses against **(A)** NDO-HSA, **(B)** LID-1, and **(C)** NDO-LID in household contacts of paucibacillary (PB-C; *n* = 20) and multibacillary (MB-C; *n* = 20) leprosy patients, and in endemic (EC; *n* = 18) and non-endemic (NEC; *n* = 20) controls. The horizontal bars represent median OD determined by ELISA. * = *P* < 0.05.

Although not differing from each other, the multibacillary (MB-C) and paucibacillary (PB-C) household contact groups displayed higher reactivity to NDO-HSA in comparison to NEC, but not when compared to EC. In addition, the EC group showed greater reactivity to NDO-HSA than NEC (Figure 2A). We did not observe significant differences in IgA reactivities to LID-1 and NDO-LID between MB-C and PB-C, or between the leprosy patient groups and controls (Figures 2B,C).

## Discussion

Despite being preventable and curable, leprosy remains a great challenge, particularly in countries such as Brazil, India, and Indonesia (23). Difficulties in early diagnosis of asymptomatic forms and of new cases represent major obstacles in controlling the disease (24). In this regard, both the inefficient surveillance systems and the reduced sensitivity of diagnostic tests have facilitated the persistence of the disease. Tests that use antigenic targets to quantify specific antibodies are being developed in efforts to accelerate diagnosis and improve leprosy control. The salient finding of this study is that patients with the multibacillary (MB) form presented high IgA reactivity to NDO-HSA, LID-1 and NDO-LID antigens. Furthermore, our study shows that, although MB patients display a more robust serum IgA response than the paucibacillary (PB) group, elevated serum IgA responses to NDO-HSA were detected regardless of the clinical form of the disease. Finally, our data indicate that household contacts diplayed higher IgA reactivity to NDO-HSA in comparison to non-endemic controls (NEC).

The phenolic glycolipid I (PGL-I) has been the main antigen used in the serological diagnosis of leprosy because increased serum IgM titers against this antigen correlate with the MB disease (25). However, other target antigens have been shown to provide a better diagnosis in early stages and in PB individuals (26). Among the several alternative antigens which have gained importance in the late years, our group and others have studied NDO-HSA, LID-1 and NDO-LID (9, 12, 27). Our previous results evaluating IgM reactivity have shown an intense response to NDO-HSA and NDO-LID in MB patients, predominantly due to the presence of NDO, a synthetic mimetic of PGL-I. The protein antigen LID-1, either alone or in association with NDO, is recognized by a robust IgG response (9). Interestingly, our experiments show that the IgA responses did not rely on the nature of the antigen, because all the evaluated antigens led to increased IgA reactivity. In that sense, it has already been demonstrated that IgA reactivity to PGL-I correlates with IgM responses in both serum and saliva (19, 28). As the nasal mucosal surfaces are the main portal of entrance for *M. leprae* (18), the antigen challenge induces local humoral antibody responses, mainly IgA, which appears early in saliva than in serum (18). Moreover, the presence of an IgA response to non-protein antigens as NDO can be justified by T-cell independent responses which are well established in the mucosa (29–31).

IgA antibodies to *M. leprae* are found in saliva and blood. Van Hooij & Geluk (2021) demonstrated that contacts of untreated leprosy patients show higher salivary IgA levels in response to either LAM or PGL-I than endemic controls (6). De Macedo et al. strongly recommended anti-PGL-I IgA as a biomarker adjunct to anti-PGL-I IgM for serological and clinical follow-up studies of household leprosy contacts in high endemical areas (19). Their results demonstrated better performance of IgA than IgG isotype by comparing the correlation of both with IgM responses. In this context, serum IgG presented low diagnostic sensitivity even in MB patients while diagnosis sensitivity based upon IgA was higher, but still far from that of serum IgM in MB patients (19).

According to Nahas et al. IgA can be a marker of exposure to *M. leprae* because of the presence of salivary IgA (sIgA) against the native LAM antigen in leprosy patients and their contacts (4). Patients with MB leprosy and with positive anti-LAM sIgA presented chances fourfold higher to develop leprosy reactions (4), the main cause of irreversible neuropathy and anatomical deformities associated with leprosy (32). Among reactional patients, 69.4% were also anti-LAM positive at diagnosis, with a 2.33-fold higher chance of developing reactions (4). The authors suggest that multidrug therapy (MDT) reduces the bacillary load and reduces anti-LAM sIgA in saliva in most patients, except in those that presented leprosy reactions (4). Although there is no accurate diagnostic test to reliably detect or predict leprosy reactions nowadays, specific antibody levels at diagnosis of leprosy could represent correlation with the risk for these reactions (32). Amorim et al. showed that MB patients who developed erythema nodosum leprosum (ENL) had increased levels of serum anti-NDO-LID IgM and IgG1 at leprosy diagnosis in comparison to MB patients who developed reversal reaction (RR) or no reaction (33). In addition, elevated anti-NDO-LID antibodies were found in people at leprosy diagnosis who went on to develop RR or ENL in the next 2 years (33).

Our study shows that, despite of the pronounced IgA response in the MB group, there was a mild response in PB group, which reflects the already well-established profile of this group, in which cellular responses overcome the humoral profile (34). Nevertheless, serum NDO-HSA IgA reactivity in PB group was higher than the control group, reaching 63% of seropositivity. Although reactivities to NDO-HSA were low, regarding the comparison with MB individuals, this results still stand out in a scenario with sensitivities for PB individual being as low as 29.2% for ELISA (35).

The antigens NDO-HSA, LID-1 and NDO-LID have been also used in surveillance studies targeting household contacts (36, 37). Leprosy household contacts represent a group at high risk of developing the disease, thus, tracking new cases among this population contributes for early detection and better control of the disease (38, 39). Whereas many authors refer to anti-PGL-I IgM as the main serological parameter in leprosy, others suggest that serum anti-PGL-I IgG/IgM and salivary anti-PGL-I IgA/IgM measurements ought to be used to follow leprosy household contacts (40). Furthermore, individuals seropositive for anti-PGL-I antibodies have a 7.5-fold greater risk of acquiring leprosy compared to seronegative contacts (40). On that way, our results indicate IgA reactivity to NDO-HSA is increased in household contacts of both paucibacillary and multibacillary groups, as well as in the endemic control in comparison to the non-endemic controls, suggesting some level of response in those groups. Nevertheless, the discrimination of household contacts based on the bacilloscopy does not seem to play any role in their IgA reactivity, as observed for NDO-LID and NDO-HSA IgM and IgG serology, elsewhere (36).

In conclusion, our data indicate that serum IgA can be used as a complementary marker of MB leprosy, mainly when focused on the NDO-HSA antigen. Distinct from IgM and IgG antibodies, IgA appears to provide good performance for MB leprosy regardless of the antigen nature. This could contribute for future diagnostic tools using broad antigen sets. Regardless, additional studies are necessary to further evaluate the potential of using IgA in the long-term serological surveillance of household contacts.

## Data Availability Statement

The raw data supporting the conclusions of this article will be made available by the authors, without undue reservation.

## Ethics Statement

The studies involving human participants were reviewed and approved by Ethical Committee of the Oswaldo Cruz Institute (protocol: 1.896.348). The patients/participants provided their written informed consent to participate in this study.

## Author Contributions

HT, MD, and ES: study conception and design. KS, EO, CE, and HT: wrote the initial draft of the manuscript. KS, IP, CE, and EO: performed the ELISA experiments and acquired and interpreted the ROC data. RP: clinical assessment and acquisition of serum samples. MD and KS: performed antigen preparation. All authors contributed to the analysis and interpretation of the data and revised the manuscript.

## Conflict of Interest

MD is employed by the company HDT Bio. The remaining authors declare that the research was conducted in the absence of any commercial or financial relationships that could be construed as a potential conflict of interest.

## Publisher's Note

All claims expressed in this article are solely those of the authors and do not necessarily represent those of their affiliated organizations, or those of the publisher, the editors and the reviewers. Any product that may be evaluated in this article, or claim that may be made by its manufacturer, is not guaranteed or endorsed by the publisher.
